# Fatty Acid Metabolism and Idiopathic Pulmonary Fibrosis

**DOI:** 10.3389/fphys.2021.794629

**Published:** 2022-01-14

**Authors:** Jing Geng, Yuan Liu, Huaping Dai, Chen Wang

**Affiliations:** ^1^Department of Pulmonary and Critical Care Medicine, Center of Respiratory Medicine, China-Japan Friendship Hospital, National Clinical Research Center for Respiratory Diseases, Institute of Respiratory Medicine, Chinese Academy of Medical Sciences, Beijing, China; ^2^Graduate School of Peking Union Medical College, Chinese Academy of Medical Sciences and Peking Union Medical College, Beijing, China

**Keywords:** fatty acid synthesis, fatty acid oxidation, idiopathic pulmonary fibrosis, alveolar epithelial cell, myofibroblast, macrophage

## Abstract

Fatty acid metabolism, including the *de novo* synthesis, uptake, oxidation, and derivation of fatty acids, plays several important roles at cellular and organ levels. Recent studies have identified characteristic changes in fatty acid metabolism in idiopathic pulmonary fibrosis (IPF) lungs, which implicates its dysregulation in the pathogenesis of this disorder. Here, we review the evidence for how fatty acid metabolism contributes to the development of pulmonary fibrosis, focusing on the profibrotic processes associated with specific types of lung cells, including epithelial cells, macrophages, and fibroblasts. We also summarize the potential therapeutics that target this metabolic pathway in treating IPF.

## Introduction

Idiopathic pulmonary fibrosis (IPF) is a fatal fibrotic disorder of unknown etiology. It is usually associated with worsening respiratory symptoms, lung function decline, and limited responses to therapies. The worldwide incidence of IPF has risen steadily over time (Hutchinson et al., 2015), and disease burdens on the global healthcare system are increasing. Underlying genetic susceptibility, together with environmental insults, is believed to trigger an abnormal wound repair response, leading to the activation of a non-resolving fibrotic cascade. Mechanistic features include epithelial apoptosis, macrophages releasing pro-fibrotic mediators, and the activation of fibroblasts and myofibroblasts.

Although the underlying mechanisms of these dysregulated fibrotic responses are not completely understood, recent evidence indicates that metabolic abnormalities play a critical role. For example, altered glycolysis and glutamine metabolism were found in human lungs with severe IPF (Kang et al., 2016). Additionally, changes in lipid metabolism, specifically leading to the overproduction of profibrotic lipids such as lysophospholipids, sphingolipids, and eicosanoids, contribute to the pathogenesis of IPF [for general review, see Castelino (2012); Mamazhakypov et al. (2019)].

In this brief review, we describe the general concepts in fatty acid (FA) metabolism and the pathology of IPF. We analyze the roles of this metabolic pathway in regulating the function of specific cells and the relevant pathologic cellular responses in IPF, including pro-fibrotic phenotype changes of alveolar epithelial cells and macrophages, and fibroblast/myofibroblast activation. We also discuss potential pulmonary fibrosis therapeutics that target this pathway.

## The Fatty Acid Metabolic Pathway

**FIGURE 1:**
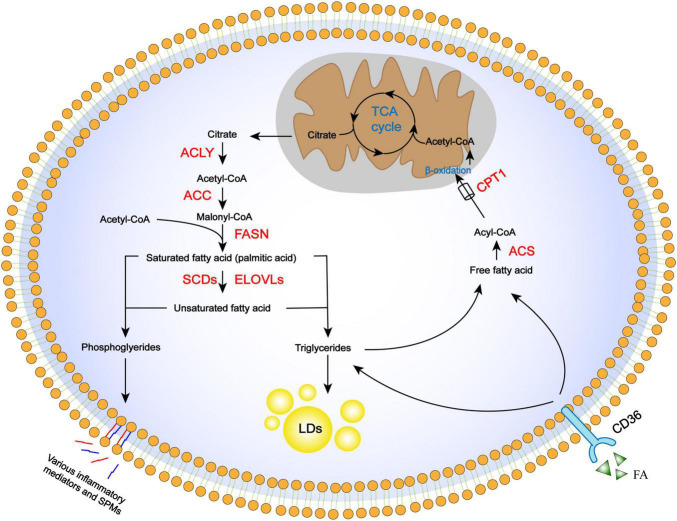
Regulation of FA metabolism pathways, including anabolism, storage, uptake, catabolism, and derivation. *De novo* FA synthesis occurs in the cytoplasm, where citrate is converted in the TCA cycle to the final long-chain saturated or unsaturated FA. These steps involve ACLY, ACC, FASN, and desaturases, as well as elongation proteins. Once synthesized, FA is stored in lipid droplets as triglycerides or mobilized through β-oxidation to provide energy and acetyl-CoA. Acetyl-CoA is used again in the TCA cycle. Essential FAs are incorporated into cellular phospholipids and released from cell membranes to be converted into lipid-derived mediators. FAs from extracellular sources can also be used for storage or β-oxidation through the CD36 receptor.

Fatty acids (FAs) contain a terminal carboxyl group and a hydrocarbon chain, and mostly contain an even number of carbons; they can be saturated or unsaturated. The understanding of the role of FA metabolism in both healthy and disease physiology has recently been greatly advanced (Kuda et al., 2018; Yi et al., 2018). Fatty acids tightly couple glucose and lipid metabolism *via* the *de novo* FA synthesis pathway, supporting cell adaption to environmental changes and generating large amounts of adenosine triphosphate (ATP) through β-oxidation (Bartlett and Eaton, 2004). As well as their role in energy production and as part of the structural “building blocks” of cell membranes, FAs also act individually by converting to FA-derived lipid mediators to regulate biological activities (de Carvalho and Caramujo, 2018) such as signal transduction, cell cycle regulation, apoptosis, and differentiation (Figure 1).

### Fatty Acid Synthesis

Fatty acid (FAs) in cells derive either from exogenous sources or *de novo* FA synthesis. The FA biosynthesis pathway is highly conserved and occurs in the cytoplasm (Rui, 2014). The TCA cycle intermediator citrate is transported out of mitochondria and cleaved by ATP citrate lyase (ACLY) into acetyl-CoA and oxaloacetate. Acetyl-CoA is then converted to malonyl-CoA by the rate-limiting enzyme acetyl-CoA carboxylase (ACC). Finally, acetyl-CoA and malonyl-CoA are used to produce palmitic acid as an initial product by the action of FA synthase (FASN) (Smith, 1994). Subsequent elongation and desaturation of palmitic acid determine the length and degree of FA saturation, which are critical to their functions and metabolic fates (Guillou et al., 2010). For example, stearate, a long-chain fatty acid, is produced through the actions of a family of enzymes, ELOVL1–7, that add two carbons to the terminal carboxyl group in each reaction cycle. Another family of stearoyl-CoA desaturases (SCDs) catalyzes FA desaturation (Paton and Ntambi, 2009). Fatty acids can also be taken up from extracellular surroundings *via* cell surface receptors, such as CD36 (Wang and Li, 2019), which is a widely expressed transmembrane protein. Once entering the intracellular FA pool, they can be esterified with glycerol or sterol backbones and stored in the form of triglycerides in lipid droplets, or utilized for energy production through FA oxidation (FAO).

### Fatty Acid Oxidation

FA oxidation (FAO) is a major pathway for the utilization of FAs to generate biological energy. Free FAs in the cytosol are activated by acyl-CoA synthase to generate acyl-CoA. Acyl-CoA is conjugated to carnitine *via* carnitine palmitoyl transferase 1 (CPT1) activity to form acylcarnitine which is subsequently transported into the mitochondrial matrix by carnitine acylcarnitine translocase (CAT). Acylcarnitine is then converted back to acyl-CoA and carnitine by CPT2 in the mitochondria. Acyl-CoA undergoes β-oxidation, which is a series of enzyme-mediated reactions that yield large amounts of intermediator metabolites that are subsequently utilized in the TCA cycle; carnitines are recycled by being transported out of the mitochondrial matrix (Houten et al., 2016).

### Fatty Acid Derivation and Derivatives

Besides anabolism and catabolism pathways in the cytoplasm, some FAs serve as substrates for enzymatic conversation to lipid-derived mediators that are bioactive in tissue inflammation and organ injury. Arachidonic acid is incorporated into cellular phospholipids and is rapidly released from cell membranes by phospholipase A2 enzymes for enzymatic conversion to prostaglandins (PGs) and leukotrienes (Kuehl and Egan, 1980) as well as lipoxins (LXs) (Serhan and Savill, 2005). Prostaglandins and leukotrienes are widely recognized for their important role in injury and inflammation, while LXs, as a family of special pre-resolving mediators (SPMs), are formed by transcellular biosynthesis and have anti-inflammatory and pro-resolving effects. These derivatives underlie the pathology of many prevalent diseases resulting in tissue fibrosis, as typified by kidney (Brennan et al., 2017), liver (Mariqueo and Zúñiga-Hernández, 2020), and lung fibrosis (Bozyk and Moore, 2011; Suryadevara et al., 2020).

## Alterations in Fatty Acid Metabolism in Pulmonary Fibrosis

An altered content and profile of saturated and unsaturated FAs have been identified in IPF patients and animal models of lung fibrosis; however, there is no consensus on the specific changes. One study observed a 63% increase in serum total FA levels in IPF patients compared with control subjects (Iannello et al., 2002). Others found low levels of saturated long-chain FAs, such as palmitic acid, oleic acid, and stearic acid, in IPF lung tissues and bronchoalveolar lavage fluid (BALF) (Schmidt et al., 2002; Kim et al., 2021). In contrast, Chu et al. (2019) detected significantly higher levels of palmitic and stearic acid in BALF from IPF patients compared with controls.

The chemotherapeutic drug bleomycin is widely used as a means of inducing experimental lung fibrosis in animal models (Fleischman et al., 1971; Adamson and Bowden, 1974; Thrall et al., 1979), including mice, rats, and dogs. Lower levels of free FAs were detected in BALF from rats exposed to bleomycin the previous day, which then reached twice normal levels on days 3–30 before returning to normal on day 120 (Swendsen et al., 1996); however, unsaturated FAs were significantly increased compared with controls between day 3 and 120 (Swendsen et al., 1996). Moreover, abnormal FA compositions were identified in the lung tissue of mice exposed to bleomycin, with high levels of palmitic acid and oleic acid but low levels of the essential polyunsaturated linoleic acid (Sunaga et al., 2013).

## Aberrant Fatty Acid Metabolism Contributes to Idiopathic Pulmonary Fibrosis Pathogenesis

**FIGURE 2:**
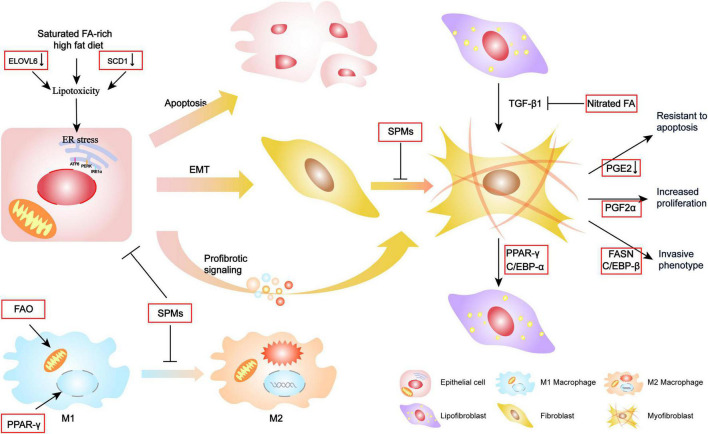
Mechanism of FA metabolism contributing to the pathogenesis of IPF. Alterations in FA metabolism contribute epithelial cellular ER stress toward apoptosis, EMT, or secretion with profibrotic signaling, which further activates fibroblast differentiation. Increasing FAO and activation of PPAR-γ transcription factor may facilitate the transportation of some M2 macrophage target genes and promote M2 polarization. SPMs could protect these cellular endophenotypes changes and fibrotic molecular signature. Additionally, FA and the regulators are also involved in the reversible lipogenic-to-myogenic differentiation and directly contribute to myofibroblast proliferation, invasion, and resistance to apoptosis.

Extensive changes in FA metabolism have been observed in IPF, suggesting its potential critical role in disease pathophysiology. Various FA metabolism pathways are intricately intertwined, and a perturbation of any of these in the lung may contribute to the development of pro-fibrotic phenotypes in epithelial cells, macrophages, and fibroblasts/myofibroblasts (Figure 2).

### Impact on Epithelial Cell Switch to a Pro-fibrotic Phenotype

Epithelial cell dysfunction is a central component in IPF pathophysiology. Alveolar type (AT)2 cells are the most active cell of lung lipid metabolism, with an important role in alveolar homeostasis involving surfactant biosynthesis, and as progenitor cells to both self-renew and transdifferentiate into AT1 cells (Barkauskas et al., 2013). AT2 cells, and other lung epithelia, are often susceptible to injury. Thus, repeated genetic or environment stimulation may result in a diverse range of cellular endophenotypes and molecular signatures including endoplasmic reticulum (ER) stress, apoptosis, and inflammatory and profibrotic signaling, which ultimately converge to drive downstream fibrotic remodeling in IPF lungs (Katzen and Beers, 2020).

Fatty acid (FA) synthesis and composition are known to be involved in ER stress (Volmer et al., 2013; Han and Kaufman, 2016; Velázquez et al., 2016). Lipids are required for the export of folded proteins contained in lipid droplets from the ER lumen (Velázquez et al., 2016), and impaired lipid synthesis can increase protein accumulation, resulting in sustained ER stress. Additionally, changes in the ER membrane composition following increased levels of FA saturation may directly activate protein kinase R-like ER kinase and inositol-requiring enzyme 1 (Volmer et al., 2013). Although the mechanism that connects FA metabolism with ER stress in IPF is unclear, studies have reported that lipotoxicity caused by saturated FA accumulation may increase ER stress, leading to apoptosis. For example, Romero et al. (2018) reported that SCD1 expression was reduced in IPF lung tissues by showing that a pharmacological inhibitor of SCD1 induced epithelial cell injury and promoted lung fibrosis by blocking the synthesis of unsaturated FAs. Moreover, a high-fat diet rich in saturated FAs has consistently been shown to induce lung epithelial cell apoptosis by causing ER stress and thereby aggravating bleomycin-induced lung fibrosis (Chu et al., 2019). Additionally, Sunaga et al. (2013) found that ELOVL6 expression was downregulated in the lungs of IPF patients. ELOVL6 catalyzes the elongation of C16 FA and renders it an unsaturated FA (Matsuzaka et al., 2007). Thus, an ELOVL6 deficiency increases the proportion of saturated FAs, thereby worsening pulmonary fibrosis with collagen deposition. Furthermore, treatment with palmitic acid was shown to trigger apoptosis and transforming growth factor (TGF)-β1 expression in cultured AT2 cells (Sunaga et al., 2013).

Although the role of the epithelial–mesenchymal transition (EMT) in IPF remains controversial, recent evidence indicates that multiple inflammatory mediators and essential FA-derived SPMs involved in EMT also function in lung fibrosis. For example, maresin 1 (MaR1), an SPM derived from docosahexaenoic acid, was found to inhibit TGF-β1-induced EMT and prevent the activation of Smad2/3, Akt, and the transcription factor Snail. Additionally, MaR1 treatment attenuated bleomycin-induced lung fibrosis *in vivo* and reduced the generation of TGF-β1 (Wang et al., 2015). Similarly, protein DX, which also derives from docosahexaenoic acid, was reported to suppress inflammatory infiltration and the expression of pro-fibrotic cytokines, and to inhibit the EMT phenotype, which prolonged the survival time of lung fibrosis mice (Li et al., 2017). Other work found that SPMs inhibited apoptosis and promoted wound repair proliferation and the transdifferentiation of AT2 cells in respiratory distress syndrome-related lung fibrosis (Zheng et al., 2018; Yang et al., 2019).

### Modulation of Macrophage Polarization

As the most abundant immune cell in the lung, macrophages play a vital role in the pathogenesis of pulmonary fibrosis. Most resident macrophages originate from progenitors in the bone marrow and migrate into different tissues where the local environment and signal cues shape the macrophage phenotype (Shapouri-Moghaddam et al., 2018). Macrophage polarization achieves a phenotypic dichotomy of the pro-inflammatory M1 subtype, which is induced by the Th1 cytokine interferon-γ, and the M2 phenotype, which is induced by the Th2 cytokines interleukin (IL)-4 or IL-13. The M2 phenotype is associated with tissue remodeling and repair and is a vital regulator of fibrogenesis in IPF (Zhang et al., 2018). Upon activation, M2 macrophages produce profibrotic mediators such as TGF-β1, which activates fibroblasts and extracellular matrix (ECM) deposition.

There appears to be a strong relationship between macrophage polarization and its demand for FAs. Fatty acids provide energy to support macrophage polarization and activate signaling pathways to shape it, while FAO generates large quantities of ATP that is thought to promote M2 polarization. Indeed, increased levels of FAO were found in IPF lungs (Gu et al., 2019), suggesting that FAO may be involved in fibrogenesis by promoting M2 macrophage activation. The M2 phenotype is also dependent upon the transcription factor peroxisome proliferator-activated receptor (PPAR)-γ (Namgaladze and Brüne, 2016), which has numerous FAs as its natural ligands (Marion-Letellier et al., 2016). Furthermore, FAs promote expression of the FA receptor CD36, thereby inducing the M2 phenotype *via* the simultaneous escalation of FA uptake and a cycle of self-augmented profibrotic activation. The loss of CD36 was reported to inhibit lung fibrosis and reduce levels of pro-fibrotic Th2 cytokines including IL-9, IL-4, and IL-13 (Parks et al., 2013). Lipoxins also regulate different macrophage subtypes in lung fibrosis, while an aspirin-triggered LX synthetic analog was reported to have the therapeutic ability to decrease cytokine production and restore M2 macrophage populations in established bleomycin-induced lung fibrosis (Martins et al., 2009; Guilherme et al., 2013).

### Activation of Fibroblasts/Myofibroblasts

Dysfunctional epithelial cells and polarized macrophages generate large quantities of profibrotic cytokines that induce fibroblast differentiation into myofibroblasts. Myofibroblasts within IPF lungs have a pathologic phenotype (Richeldi et al., 2017), including the ability to secrete excessive amounts of matrix within lung parenchyma, and causing basement membrane disruption. The main source of lung myofibroblasts is resident interstitial lung fibroblasts. Recently, lipofibroblast was suggested as an origin of the activated myofibroblast in pulmonary fibrosis (El Agha et al., 2017). Lipofibroblast is one of the interstitial fibroblasts that contain lipid droplets and are located adjacent to AT2 cells, and likely contribute to surfactant production in quiescent lungs (Rehan and Torday, 2014).

El Agha et al. (2017) used cell lineage tracing to monitor lipogenic or myogenic populations of lung fibroblasts in mice and demonstrated a phenotypic switch between the two populations during the progression and resolution phases of lung fibrosis. Mechanically, they found that this phenotypic shift involves PPAR-γ. Nitrated fatty acids are PPAR-γ agonists which promote the dedifferentiation of myofibroblasts by blocking TGF-β1 effects (Reddy et al., 2014). Additionally, CCAAT enhancer-binding protein (C/EBP) α, which is regulated by FAs and their derivatives in adipogenesis, was found to promote myofibroblast to lipofibroblast dedifferentiation (Liu et al., 2019). Both non-fibrotic controls and IPF-derived fibroblasts/myofibroblasts express LXA4 receptors that enable activation of the ALXR G-protein-coupled receptor to regress a myofibroblastic phenotype to a fibroblastic one by reducing α-SMA expression, actin stress fiber formation, and nuclear Smad2/3 levels (Roach et al., 2015).

Additional characteristic phenotypes of fibroblasts/myofibroblasts that contribute to the development of lung fibrosis include increased proliferation, resistance to apoptosis, and the acquisition of invasive activity (Hinz and Lagares, 2020; Phan et al., 2021). During wound healing, fibroblasts proliferate in response to tissue injury but eventually disappear through apoptosis when the tissue returns to homeostasis. However, IPF fibroblasts demonstrate increased proliferation and resistance to FAS ligand-induced apoptosis (Bamberg et al., 2018). Idiopathic pulmonary fibrosis lungs have also been shown to have an abundance of PGF2α that stimulates the proliferation of lung fibroblasts (Oga et al., 2009), while IPF-derived fibroblasts have reduced PGE2 levels that contribute to their apoptotic resistance (Maher et al., 2010). Indeed, PGE2 limits many of the pathologic features of lung fibroblasts and myofibroblasts, including proliferation, migration, collagen secretion, and TGFβ1-induced differentiation (reviewed in Bozyk and Moore, 2011). These findings suggest that prostaglandins have pleiotropic activities in regulating the fibroblast phenotype in IPF.

Unlike normal fibroblasts, IPF fibroblasts invade the surrounding ECM much like metastatic cancer cells (Ballester et al., 2019). The underlying mechanism for this enhanced invasion may be correlated with *de novo* FA synthesis because the inhibition of *FASN* attenuated the invasive activity of TGFβ1-treated fibroblasts (Jung et al., 2018). Another possibility is the formation of α-smooth muscle actin (α-SMA)-containing stress fibers. A cognate binding element of C/EBPβ was identified in the α*-SMA* promoter that contributed to its upregulation (Phan, 2012).

## Potential Therapeutics Targeting the Fatty Acid Metabolic Pathway in Idiopathic Pulmonary Fibrosis Treatment

Therapies targeting FA metabolism have been tested in pre-clinical models of lung fibrosis (Table 1). Promoting the conversion and formation of FAs with small molecule compounds, such as a liver X receptor agonist (T0901317) and PPAR-γ agonists (e.g., rosiglitazone, pioglitazone, and troglitazone), was found to be beneficial in animal models of lung fibrosis (Genovese et al., 2005; Milam et al., 2008; Aoki et al., 2009; Romero et al., 2018). Metformin also exerts potent antifibrotic effects, including altering the fate of myofibroblasts *via* PPAR-γ activation and inhibiting collagen production *via* AMP-activated protein kinase activation (Kheirollahi et al., 2019; Xiao et al., 2020). Based on the altered content and composition of FAs in IPF lungs and animal models that we describe, it seems a reasonable approach to supplement appropriate FAs for the treatment of this disease. Indeed, dietary essential FAs provide protection from lung fibrosis after bleomycin treatment (Kennedy et al., 1989), while intratracheally delivered FAs have a therapeutic potential for the treatment of lung fibrosis (Zhao et al., 2014).

**TABLE 1:** Summary of studies evaluating the effects of fatty acid-targeting agents in animal models of lung fibrosis.

Agent	Target and mechanism	Strategy	Animal models	Agent application	Main effects	References
T0901317	Augment expression of several lipid-synthesizing enzyme	curative	Silica, mice (14 days)	Intraperitoneal, daily (days 4–14)	Reduce ER stress and attenuate fibrotic remodeling	Romero et al., 2018
Troglitazone	Activation of the PPAR-r signaling	curative preventive	Bleomycin, mice (21days)	Oral, daily (days -3–21 or days 11–21)	Reduce fibrosis and TGF-β1 levels	Jung et al., 2018
Pioglitazone	Activation of the PPAR-r signaling	preventive	Bleomycin, rats (28 days)	Oral, daily (days -7–28)	Prevent inflammation and collagen synthesis	Genovese et al., 2005
Metformin	Activation of the AMPK and PPAR-r signaling	curative	Bleomycin, mice (28 days)	Supplied *via* drinking water (days 14–28)	Induce lipogenic differentiation in myofibroblast and accelerate resolution of fibrosis	Kheirollahi et al., 2019
Docosahexa-enoic acid	A single n-3 polyunsaturated fatty acid	preventive	Bleomycin, mice (21 days)	Intratracheal, once (days -4–21)	Reduce weight loss and mortality; reduce fibrosis; reduce lung function changes	Kennedy et al., 1989
Dietary essential fatty acids	Rich in omega-3 fatty acid, eicosapentaenoic acid, and docosahexaenoic acid	preventive	Bleomycin, mice (21 days)	Dietary treatment began at 21 days of age and continued for the entire study	Reduce the severity of fibrosis	Zhao et al., 2014

*ER, endoplasmic reticulum; PPAR-γ, peroxisome proliferator-activated receptor γ; TGF-β1, transforming growth factor β1, AMPK, AMP-activated protein kinase, ROS, reactive oxidative species; ATP, adenosine triphosphate.*

## Conclusion

Abnormalities of FA metabolism in pulmonary fibrosis have received increasing attention in recent years. This review describes how the FA metabolism regulates the profibrotic phenotype of alveolar epithelial cells and macrophages, as well as fibroblasts/myofibroblasts activation in the IPF lungs and the lungs of mice with experimental pulmonary fibrosis. Understanding the mechanism of these metabolic abnormalities in IPF will open a new avenue of novel therapeutics.

## Author Contributions

All authors contributed to the writing of the article and the development of the figures.

## Conflict of Interest

The authors declare that the research was conducted in the absence of any commercial or financial relationships that could be construed as a potential conflict of interest.

## Publisher’s Note

All claims expressed in this article are solely those of the authors and do not necessarily represent those of their affiliated organizations, or those of the publisher, the editors and the reviewers. Any product that may be evaluated in this article, or claim that may be made by its manufacturer, is not guaranteed or endorsed by the publisher.

## References

[B1] Adamson I. Y. Bowden D. H. (1974). The pathogenesis of bleomycin-induced pulmonary fibrosis in mice. *Am. J. Pathol.* 77 185–197. 4141224 PMC1910906

[B2] Aoki Y. Maeno T. Aoyagi K. Ueno M. Aoki F. Aoki N. (2009). Pioglitazone, a peroxisome proliferator-activated receptor gamma ligand, suppresses bleomycin-induced acute lung injury and fibrosis. *Respiration* 77 311–319. 10.1159/000168676 18974632

[B3] Ballester B. Milara J. Cortijo J. (2019). Idiopathic pulmonary fibrosis and lung cancer: mechanisms and molecular targets. *Int. J. Mol. Sci.* 20:593. 10.3390/ijms20030593 30704051 PMC6387034

[B4] Bamberg A. Redente E. F. Groshong S. D. Tuder R. M. Cool C. D. Keith R. C. (2018). Protein tyrosine phosphatase-N13 promotes myofibroblast resistance to apoptosis in idiopathic pulmonary fibrosis. *Am. J. Respir. Crit. Care Med.* 198 914–927. 10.1164/rccm.201707-1497OC 29727583 PMC6173065

[B5] Barkauskas C. E. Cronce M. J. Rackley C. R. Bowie E. J. Keene D. R. Stripp B. R. (2013). Type 2 alveolar cells are stem cells in adult lung. *J. Clin. Invest.* 123 3025–3036. 10.1172/jci68782 23921127 PMC3696553

[B6] Bartlett K. Eaton S. (2004). Mitochondrial beta-oxidation. *Eur. J. Biochem.* 271 462–469. 10.1046/j.1432-1033.2003.03947.x 14728673

[B7] Bozyk P. D. Moore B. B. (2011). Prostaglandin E2 and the pathogenesis of pulmonary fibrosis. *Am. J. Respir. Cell Mol. Biol.* 45 445–452. 10.1165/rcmb.2011-0025RT 21421906 PMC3175580

[B8] Brennan E. P. Cacace A. Godson C. (2017). Specialized pro-resolving mediators in renal fibrosis. *Mol. Aspects Med.* 58 102–113. 10.1016/j.mam.2017.05.001 28479307

[B9] Castelino F. V. (2012). Lipids and eicosanoids in fibrosis: emerging targets for therapy. *Curr. Opin. Rheumatol.* 24 649–655. 10.1097/BOR.0b013e328356d9f6 22810365

[B10] Chu S. G. Villalba J. A. Liang X. Xiong K. Tsoyi K. Ith B. (2019). Palmitic acid-rich high-fat diet exacerbates experimental pulmonary fibrosis by modulating endoplasmic reticulum stress. *Am. J. Respir. Cell Mol. Biol.* 61 737–746. 10.1165/rcmb.2018-0324OC 31461627 PMC6890409

[B11] de Carvalho C. Caramujo M. J. (2018). The various roles of fatty acids. *Molecules* 23:2583. 10.3390/molecules23102583 30304860 PMC6222795

[B12] El Agha E. Moiseenko A. Kheirollahi V. De Langhe S. Crnkovic S. Kwapiszewska G. (2017). Two-way conversion between lipogenic and myogenic fibroblastic phenotypes marks the progression and resolution of lung fibrosis. *Cell Stem Cell* 20 261–273.e3. 10.1016/j.stem.2016.10.004 27867035 PMC5291816

[B13] Fleischman R. W. Baker J. R. Thompson G. R. Schaeppi U. H. Illievski V. R. Cooney D. A. (1971). Bleomycin-induced interstitial pneumonia in dogs. *Thorax* 26 675–682. 10.1136/thx.26.6.675 4111464 PMC472381

[B14] Genovese T. Cuzzocrea S. Di Paola R. Mazzon E. Mastruzzo C. Catalano P. (2005). Effect of rosiglitazone and 15-deoxy-Delta12,14-prostaglandin J2 on bleomycin-induced lung injury. *Eur. Respir. J.* 25 225–234. 10.1183/09031936.05.00049704 15684285

[B15] Gu L. Larson Casey J. L. Andrabi S. A. Lee J. H. Meza-Perez S. Randall T. D. (2019). Mitochondrial calcium uniporter regulates PGC-1α expression to mediate metabolic reprogramming in pulmonary fibrosis. *Redox Biol.* 26:101307. 10.1016/j.redox.2019.101307 31473487 PMC6831865

[B16] Guilherme R. F. Xisto D. G. Kunkel S. L. Freire-de-Lima C. G. Rocco P. R. Neves J. S. (2013). Pulmonary antifibrotic mechanisms aspirin-triggered lipoxin A(4) synthetic analog. *Am. J. Respir. Cell Mol. Biol.* 49 1029–1037. 10.1165/rcmb.2012-0462OC 23848293 PMC5459549

[B17] Guillou H. Zadravec D. Martin P. G. Jacobsson A. (2010). The key roles of elongases and desaturases in mammalian fatty acid metabolism: insights from transgenic mice. *Prog. Lipid Res.* 49 186–199. 10.1016/j.plipres.2009.12.002 20018209

[B18] Han J. Kaufman R. J. (2016). The role of ER stress in lipid metabolism and lipotoxicity. *J. Lipid Res.* 57 1329–1338. 10.1194/jlr.R067595 27146479 PMC4959874

[B19] Hinz B. Lagares D. (2020). Evasion of apoptosis by myofibroblasts: a hallmark of fibrotic diseases. *Nat. Rev. Rheumatol.* 16 11–31. 10.1038/s41584-019-0324-5 31792399 PMC7913072

[B20] Houten S. M. Violante S. Ventura F. V. Wanders R. J. (2016). The biochemistry and physiology of mitochondrial fatty acid β-oxidation and its genetic disorders. *Annu. Rev. Physiol.* 78 23–44. 10.1146/annurev-physiol-021115-105045 26474213

[B21] Hutchinson J. Fogarty A. Hubbard R. McKeever T. (2015). Global incidence and mortality of idiopathic pulmonary fibrosis: a systematic review. *Eur. Respir. J.* 46 795–806. 10.1183/09031936.00185114 25976683

[B22] Iannello S. Cavaleri A. Camuto M. Pisano M. G. Milazzo P. Belfiore F. (2002). Low fasting serum triglyceride and high free fatty acid levels in pulmonary fibrosis: a previously unreported finding. *MedGenMed* 4:5. 12145565

[B23] Jung M. Y. Kang J. H. Hernandez D. M. Yin X. Andrianifahanana M. Wang Y. (2018). Fatty acid synthase is required for profibrotic TGF-β signaling. *FASEB J.* 32 3803–3815. 10.1096/fj.201701187R 29475397 PMC5998981

[B24] Kang Y. P. Lee S. B. Lee J. M. Kim H. M. Hong J. Y. Lee W. J. (2016). Metabolic profiling regarding pathogenesis of idiopathic pulmonary fibrosis. *J. Proteome Res.* 15 1717–1724. 10.1021/acs.jproteome.6b00156 27052453

[B25] Katzen J. Beers M. F. (2020). Contributions of alveolar epithelial cell quality control to pulmonary fibrosis. *J. Clin. Invest.* 130 5088–5099. 10.1172/jci139519 32870817 PMC7524463

[B26] Kennedy J. I. Jr. Chandler D. B. Fulmer J. D. Wert M. B. Grizzle W. E. (1989). Dietary fish oil inhibits bleomycin-induced pulmonary fibrosis in the rat. *Exp. Lung Res.* 15 315–329. 10.3109/01902148909087861 2468480

[B27] Kheirollahi V. Wasnick R. M. Biasin V. Vazquez-Armendariz A. I. Chu X. Moiseenko A. (2019). Metformin induces lipogenic differentiation in myofibroblasts to reverse lung fibrosis. *Nat. Commun.* 10:2987. 10.1038/s41467-019-10839-0 31278260 PMC6611870

[B28] Kim H. S. Yoo H. J. Lee K. M. Song H. E. Kim S. J. Lee J. O. (2021). Stearic acid attenuates profibrotic signalling in idiopathic pulmonary fibrosis. *Respirology* 26 255–263. 10.1111/resp.13949 33025706

[B29] Kuda O. Rossmeisl M. Kopecky J. (2018). Omega-3 fatty acids and adipose tissue biology. *Mol. Aspects Med.* 64 147–160. 10.1016/j.mam.2018.01.004 29329795

[B30] Kuehl F. A. Jr. Egan R. W. (1980). Prostaglandins, arachidonic acid, and inflammation. *Science* 210 978–984. 10.1126/science.6254151 6254151

[B31] Li H. Hao Y. Zhang H. Ying W. Li D. Ge Y. (2017). Posttreatment with Protectin DX ameliorates bleomycin-induced pulmonary fibrosis and lung dysfunction in mice. *Sci. Rep.* 7:46754. 10.1038/srep46754 28466866 PMC5413938

[B32] Liu W. Meridew J. A. Aravamudhan A. Ligresti G. Tschumperlin D. J. Tan Q. (2019). Targeted regulation of fibroblast state by CRISPR-mediated CEBPA expression. *Respir. Res.* 20:281. 10.1186/s12931-019-1253-1 31829168 PMC6907247

[B33] Maher T. M. Evans I. C. Bottoms S. E. Mercer P. F. Thorley A. J. Nicholson A. G. (2010). Diminished prostaglandin E2 contributes to the apoptosis paradox in idiopathic pulmonary fibrosis. *Am. J. Respir. Crit. Care Med.* 182 73–82. 10.1164/rccm.200905-0674OC 20203246 PMC2902759

[B34] Mamazhakypov A. Schermuly R. T. Schaefer L. Wygrecka M. (2019). Lipids – two sides of the same coin in lung fibrosis. *Cell. Signal.* 60 65–80. 10.1016/j.cellsig.2019.04.007 30998969

[B35] Marion-Letellier R. Savoye G. Ghosh S. (2016). Fatty acids, eicosanoids and PPAR gamma. *Eur. J. Pharmacol.* 785 44–49. 10.1016/j.ejphar.2015.11.004 26632493

[B36] Mariqueo T. A. Zúñiga-Hernández J. (2020). Omega-3 derivatives, specialized pro-resolving mediators: promising therapeutic tools for the treatment of pain in chronic liver disease. *Prostaglandins Leukot. Essent. Fatty Acids* 158:102095. 10.1016/j.plefa.2020.102095 32450460

[B37] Martins V. Valença S. S. Farias-Filho F. A. Molinaro R. Simões R. L. Ferreira T. P. (2009). ATLa, an aspirin-triggered lipoxin A4 synthetic analog, prevents the inflammatory and fibrotic effects of bleomycin-induced pulmonary fibrosis. *J. Immunol.* 182 5374–5381. 10.4049/jimmunol.0802259 19380784

[B38] Matsuzaka T. Shimano H. Yahagi N. Kato T. Atsumi A. Yamamoto T. (2007). Crucial role of a long-chain fatty acid elongase, Elovl6, in obesity-induced insulin resistance. *Nat. Med.* 13 1193–1202. 10.1038/nm1662 17906635

[B39] Milam J. E. Keshamouni V. G. Phan S. H. Hu B. Gangireddy S. R. Hogaboam C. M. (2008). PPAR-gamma agonists inhibit profibrotic phenotypes in human lung fibroblasts and bleomycin-induced pulmonary fibrosis. *Am. J. Physiol. Lung Cell Mol. Physiol.* 294 L891–L901. 10.1152/ajplung.00333.2007 18162602 PMC5926773

[B40] Namgaladze D. Brüne B. (2016). Macrophage fatty acid oxidation and its roles in macrophage polarization and fatty acid-induced inflammation. *Biochim. Biophys. Acta* 1861 1796–1807. 10.1016/j.bbalip.2016.09.002 27614008

[B41] Oga T. Matsuoka T. Yao C. Nonomura K. Kitaoka S. Sakata D. (2009). Prostaglandin F(2alpha) receptor signaling facilitates bleomycin-induced pulmonary fibrosis independently of transforming growth factor-beta. *Nat. Med.* 15 1426–1430. 10.1038/nm.2066 19966781

[B42] Parks B. W. Black L. L. Zimmerman K. A. Metz A. E. Steele C. Murphy-Ullrich J. E. (2013). CD36, but not G2A, modulates efferocytosis, inflammation, and fibrosis following bleomycin-induced lung injury. *J. Lipid Res.* 54 1114–1123. 10.1194/jlr.M035352 23393303 PMC3605987

[B43] Paton C. M. Ntambi J. M. (2009). Biochemical and physiological function of stearoyl-CoA desaturase. *Am. J. Physiol. Endocrinol. Metab.* 297 E28–E37. 10.1152/ajpendo.90897.2008 19066317 PMC2711665

[B44] Phan S. H. (2012). Genesis of the myofibroblast in lung injury and fibrosis. *Proc. Am. Thorac. Soc.* 9 148–152. 10.1513/pats.201201-011AW 22802289 PMC5830705

[B45] Phan T. H. G. Paliogiannis P. Nasrallah G. K. Giordo R. Eid A. H. Fois A. G. (2021). Emerging cellular and molecular determinants of idiopathic pulmonary fibrosis. *Cell. Mol. Life Sci.* 78 2031–2057. 10.1007/s00018-020-03693-7 33201251 PMC7669490

[B46] Reddy A. T. Lakshmi S. P. Zhang Y. Reddy R. C. (2014). Nitrated fatty acids reverse pulmonary fibrosis by dedifferentiating myofibroblasts and promoting collagen uptake by alveolar macrophages. *FASEB J.* 28 5299–5310. 10.1096/fj.14-256263 25252739 PMC4232282

[B47] Rehan V. K. Torday J. S. (2014). The lung alveolar lipofibroblast: an evolutionary strategy against neonatal hyperoxic lung injury. *Antioxid. Redox Signal.* 21 1893–1904. 10.1089/ars.2013.5793 24386954 PMC4202930

[B48] Richeldi L. Collard H. R. Jones M. G. (2017). Idiopathic pulmonary fibrosis. *Lancet* 389 1941–1952. 10.1016/s0140-6736(17)30866-8 28365056

[B49] Roach K. M. Feghali-Bostwick C. A. Amrani Y. Bradding P. (2015). Lipoxin A4 attenuates constitutive and TGF-β1-dependent profibrotic activity in human lung myofibroblasts. *J. Immunol.* 195 2852–2860. 10.4049/jimmunol.1500936 26276873 PMC4560490

[B50] Romero F. Hong X. Shah D. Kallen C. B. Rosas I. Guo Z. (2018). Lipid synthesis is required to resolve endoplasmic reticulum stress and limit fibrotic responses in the lung. *Am. J. Respir. Cell Mol. Biol.* 59 225–236. 10.1165/rcmb.2017-0340OC 29465261 PMC6096342

[B51] Rui L. (2014). Energy metabolism in the liver. *Compr. Physiol.* 4 177–197. 10.1002/cphy.c130024 24692138 PMC4050641

[B52] Schmidt R. Meier U. Markart P. Grimminger F. Velcovsky H. G. Morr H. (2002). Altered fatty acid composition of lung surfactant phospholipids in interstitial lung disease. *Am. J. Physiol. Lung Cell Mol. Physiol.* 283 L1079–L1085. 10.1152/ajplung.00484.2001 12376361

[B53] Serhan C. N. Savill J. (2005). Resolution of inflammation: the beginning programs the end. *Nat. Immunol.* 6 1191–1197. 10.1038/ni1276 16369558

[B54] Shapouri-Moghaddam A. Mohammadian S. Vazini H. Taghadosi M. Esmaeili S. A. Mardani F. (2018). Macrophage plasticity, polarization, and function in health and disease. *J. Cell Physiol.* 233 6425–6440. 10.1002/jcp.26429 29319160 PMC13482560

[B55] Smith S. (1994). The animal fatty acid synthase: one gene, one polypeptide, seven enzymes. *FASEB J.* 8 1248–1259. 8001737

[B56] Sunaga H. Matsui H. Ueno M. Maeno T. Iso T. Syamsunarno M. R. (2013). Deranged fatty acid composition causes pulmonary fibrosis in Elovl6-deficient mice. *Nat. Commun.* 4:2563. 10.1038/ncomms3563 24113622

[B57] Suryadevara V. Ramchandran R. Kamp D. W. Natarajan V. (2020). Lipid mediators regulate pulmonary fibrosis: potential mechanisms and signaling pathways. *Int. J. Mol. Sci.* 21:4257. 10.3390/ijms21124257 32549377 PMC7352853

[B58] Swendsen C. L. Skita V. Thrall R. S. (1996). Alterations in surfactant neutral lipid composition during the development of bleomycin-induced pulmonary fibrosis. *Biochim. Biophys. Acta* 1301 90–96. 10.1016/0005-2760(96)00023-9 8652656

[B59] Thrall R. S. McCormick J. R. Jack R. M. McReynolds R. A. Ward P. A. (1979). Bleomycin-induced pulmonary fibrosis in the rat: inhibition by indomethacin. *Am. J. Pathol.* 95 117–130. 86304 PMC2042298

[B60] Velázquez A. P. Tatsuta T. Ghillebert R. Drescher I. Graef M. (2016). Lipid droplet-mediated ER homeostasis regulates autophagy and cell survival during starvation. *J. Cell Biol.* 212 621–631. 10.1083/jcb.201508102 26953354 PMC4792078

[B61] Volmer R. van der Ploeg K. Ron D. (2013). Membrane lipid saturation activates endoplasmic reticulum unfolded protein response transducers through their transmembrane domains. *Proc. Natl. Acad. Sci. U.S.A.* 110 4628–4633. 10.1073/pnas.1217611110 23487760 PMC3606975

[B62] Wang J. Li Y. (2019). CD36 tango in cancer: signaling pathways and functions. *Theranostics* 9 4893–4908. 10.7150/thno.36037 31410189 PMC6691380

[B63] Wang Y. Li R. Chen L. Tan W. Sun Z. Xia H. (2015). Maresin 1 inhibits epithelial-to-mesenchymal transition in vitro and attenuates bleomycin induced lung fibrosis in vivo. *Shock* 44 496–502. 10.1097/shk.0000000000000446 26196843

[B64] Xiao H. Huang X. Wang S. Liu Z. Dong R. Song D. (2020). Metformin ameliorates bleomycin-induced pulmonary fibrosis in mice by suppressing IGF-1. *Am. J. Transl. Res.* 12 940–949. 32269725 PMC7137034

[B65] Yang Y. Hu L. Xia H. Chen L. Cui S. Wang Y. (2019). Resolvin D1 attenuates mechanical stretch-induced pulmonary fibrosis *via* epithelial-mesenchymal transition. *Am. J. Physiol. Lung Cell Mol. Physiol.* 316 L1013–L1024. 10.1152/ajplung.00415.2018 30724098

[B66] Yi M. Li J. Chen S. Cai J. Ban Y. Peng Q. (2018). Emerging role of lipid metabolism alterations in Cancer stem cells. *J. Exp. Clin. Cancer Res.* 37:118. 10.1186/s13046-018-0784-5 29907133 PMC6003041

[B67] Zhang L. Wang Y. Wu G. Xiong W. Gu W. Wang C. Y. (2018). Macrophages: friend or foe in idiopathic pulmonary fibrosis? *Respir. Res.* 19:170. 10.1186/s12931-018-0864-2 30189872 PMC6127991

[B68] Zhao H. Chan-Li Y. Collins S. L. Zhang Y. Hallowell R. W. Mitzner W. (2014). Pulmonary delivery of docosahexaenoic acid mitigates bleomycin-induced pulmonary fibrosis. *BMC Pulm. Med.* 14:64. 10.1186/1471-2466-14-64 24742272 PMC3998951

[B69] Zheng S. Wang Q. D’Souza V. Bartis D. Dancer R. Parekh D. (2018). ResolvinD(1) stimulates epithelial wound repair and inhibits TGF-β-induced EMT whilst reducing fibroproliferation and collagen production. *Lab. Invest.* 98 130–140. 10.1038/labinvest.2017.114 29083412 PMC5754464

