# Supplementary material for: Fatty Acid Metabolism and Idiopathic Pulmonary Fibrosis
Source: Front Physiol. 2022 Jan 14;12:794629. doi: 10.3389/fphys.2021.794629 (PMC8795701; doi:10.3389/fphys.2021.794629)
Supplement: Supplementary file 1 [file Table_1.DOCX]

**Table 1**

Summary of studies evaluating the effects of fatty acid-targeting agents in animal models of lung fibrosis.

| Agent | Target and mechanism | strategy | Animal models | Agent application | Main effects | References |
| --- | --- | --- | --- | --- | --- | --- |
| T0901317 | Augment expression of several lipid-synthesizing enzyme | curative | Silica, mice (14 days) | Intraperitoneal, daily  (days 4-14) | Reduce ER stress and attenuate fibrotic remodeling | Romero et.al. (2018) |
| Troglitazone | Activation of the PPAR-r signaling | curative preventive | Bleomycin, mice (21days) | Oral, daily  (days -3-21 or days 11-21) | Reduce fibrosis and TGF-β1 levels | Jung et.al. (2018) |
| Pioglitazone | Activation of the PPAR-r signaling | preventive | Bleomycin, rats (28 days) | Oral, daily  (days -7-28) | Prevent inflammation and collagen synthesis | Genovese et.al. (2005) |
| Metformin | Activation of the AMPK and PPAR-r signaling | curative | Bleomycin, mice (28 days) | Supplied via drinking water  (days 14-28) | Induce lipogenic differentiation in myofibroblast and accelerate resolution of fibrosis | Kheirollahi et.al (2019) |
| Docosahexa-enoic acid | A single n-3 polyunsaturated fatty acid | preventive | Bleomycin, mice (21 days) | Intratracheal, once  (days -4-21) | Reduce weight loss and mortality; reduce fibrosis;  reduce lung function changes | Kenedy et.al (1989) |
| Dietary essential fatty acids | Rich in omega-3 fatty acid, eicosapentaenoic acid and docosahexaenoic acid | preventive | Bleomycin, mice (21 days) | Dietary treatment begun at 21 days of age and continued for the entire study | Reduce the severity of fibrosis | Zhao et.al (2014) |

ER, endoplasmic reticulum; PPAR-γ, peroxisome proliferator-activated receptor γ; TGF-β1, transforming growth factor β1, AMPK, AMP-activated protein kinase, ROS, reactive oxidative species; ATP, adenosine triphosphate
